# RETRACTED: Johnson et al. Effect of Education on Adherence to Recommended Prenatal Practices Among Indigenous Ngäbe–Buglé Communities of Panama. *Medicina* 2024, *60*, 1055

**DOI:** 10.3390/medicina62020383

**Published:** 2026-02-14

**Authors:** Sabrina M. Johnson, Erin N. Kelly, Benjamin LaBrot, Kristen Ryczak

**Affiliations:** 1 Drexel University College of Medicine, Philadelphia, PA 19104, USA; 2 Keck School of Medicine, University of Southern California, Los Angeles, CA 90033, USA; 3 Department of Family, Community, & Preventive Medicine, Drexel University College of Medicine, Philadelphia, PA 19104, USA

The journal retracts the article titled “Effect of Education on Adherence to Recommended Prenatal Practices among Indigenous Ngäbe–Buglé Communities of Panama” [1], cited above.

Following publication, concerns were brought to the attention of the Editorial Office regarding the study’s compliance with Panamanian legislation governing ethical approval requirements.

In accordance with standard journal procedures, the Editorial Office and Editorial Board conducted an investigation, with the input from the National Committee on Research Bioethics of Panama. The investigation confirmed that, while Institutional Review Board (IRB) approval was gained, approval from the National Committee on Research Bioethics, as required by national law, had not been secured prior to the commencement of this study. The authors fully cooperated with the investigation and formally requested the retraction of their publication.

As a result, the Editorial Board have decided to retract this paper as per MDPI’s retraction policy.

This retraction was approved by the Editor-in-Chief of *Medicina*.

The authors agree with this decision.
